# Supplementary material for: Distinct neural mechanisms of social orienting and mentalizing revealed by independent measures of neural and eye movement typicality
Source: Commun Biol. 2020 Jan 29;3:48. doi: 10.1038/s42003-020-0771-1 (PMC6989525; doi:10.1038/s42003-020-0771-1)
Supplement: Supplementary file 1 — Supplementary Material [file 42003_2020_771_MOESM1_ESM.docx]

**
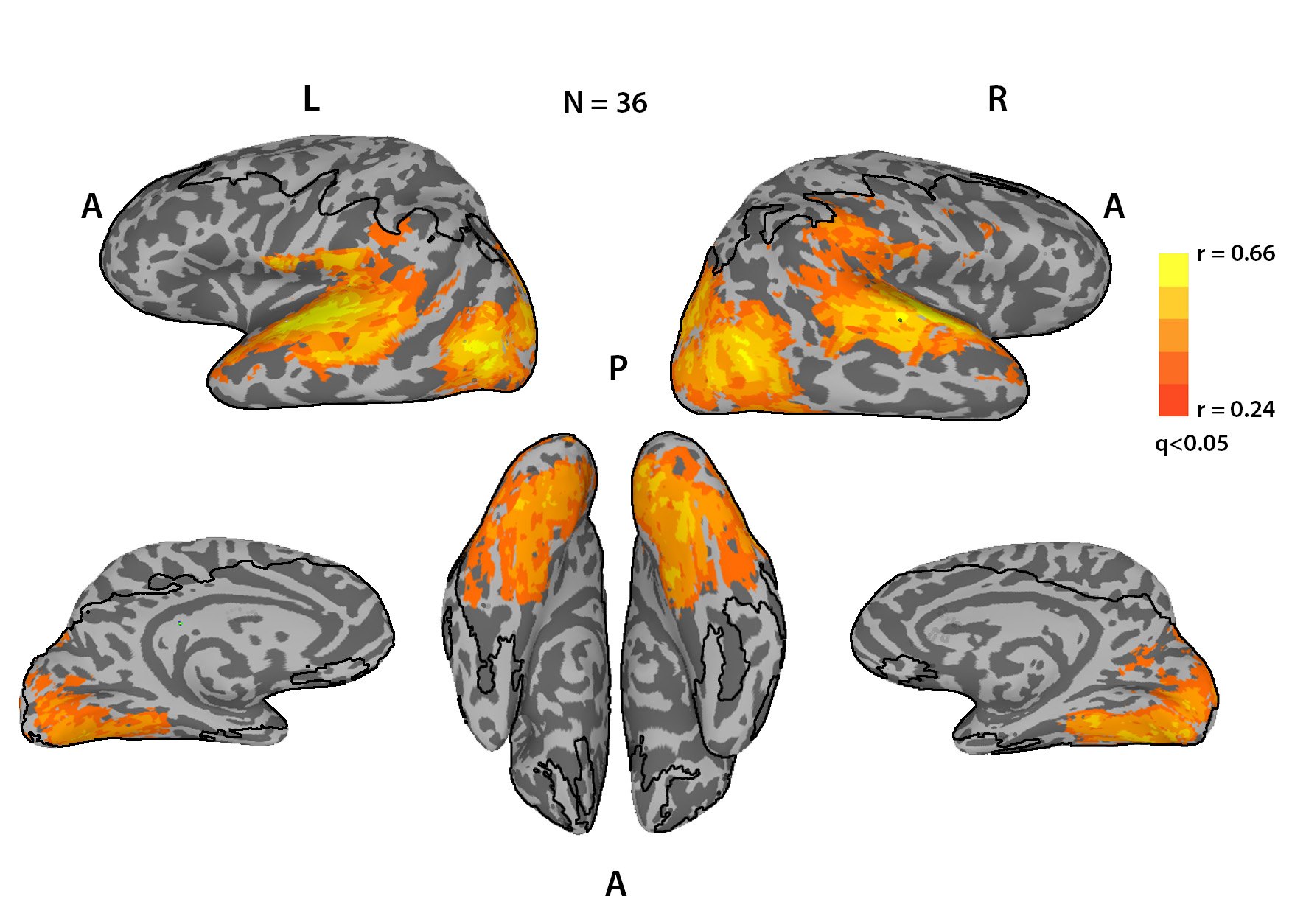
**

**Supplementary Figure 1: average neural typicality for the ASD group**. Average neural typicality (ISC) across the entire brain for the ASD participants, calculated in relation to the average typicality of the TD group and thresholded at q<0.05, FDR corrected. Black lines delineate the field of view, voxels outside this boundary were not imaged or were removed from the analysis for poor temporal signal to noise ratio (tSNR).


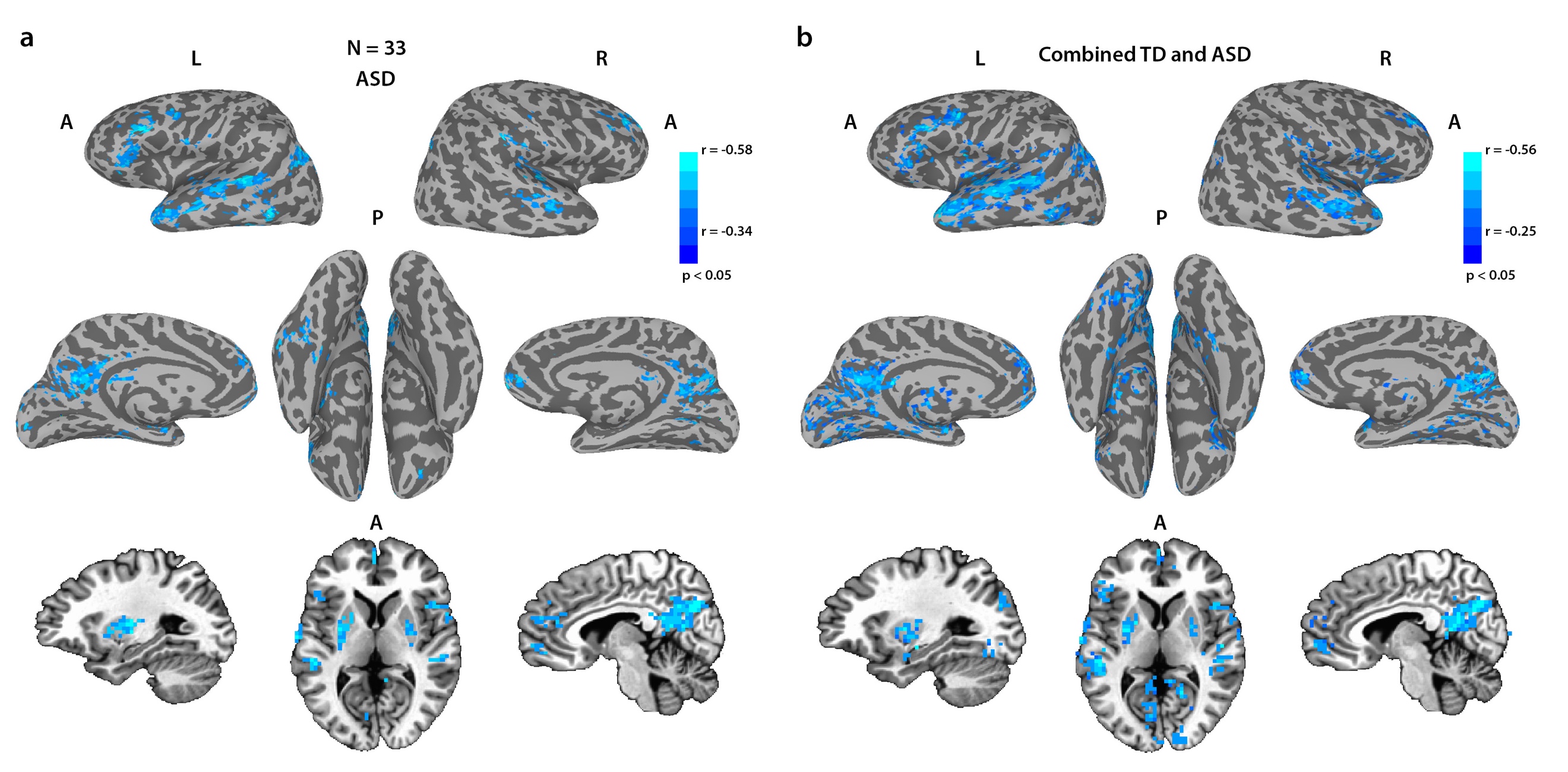


**Supplementary Figure 2: correlations between neural typicality and eye movement typicality**. (a) Correlations between eye movement typicality and neural typicality for the ASD group at a corrected threshold of p<0.05, corrected through cluster size permutation testing. (b) Correlations between eye movement typicality and neural typicality for the combined group of the ASD participants and their matched controls (N=36 ASD + 33 matched TD for a total of N=69), after subtracting the group average for both eye movement typicality and neural typicality for each group. Note the similarity of both these results to the results of the correlation between eye movement typicality and neural typicality for the full TD group, presented in Figure 4a.

**
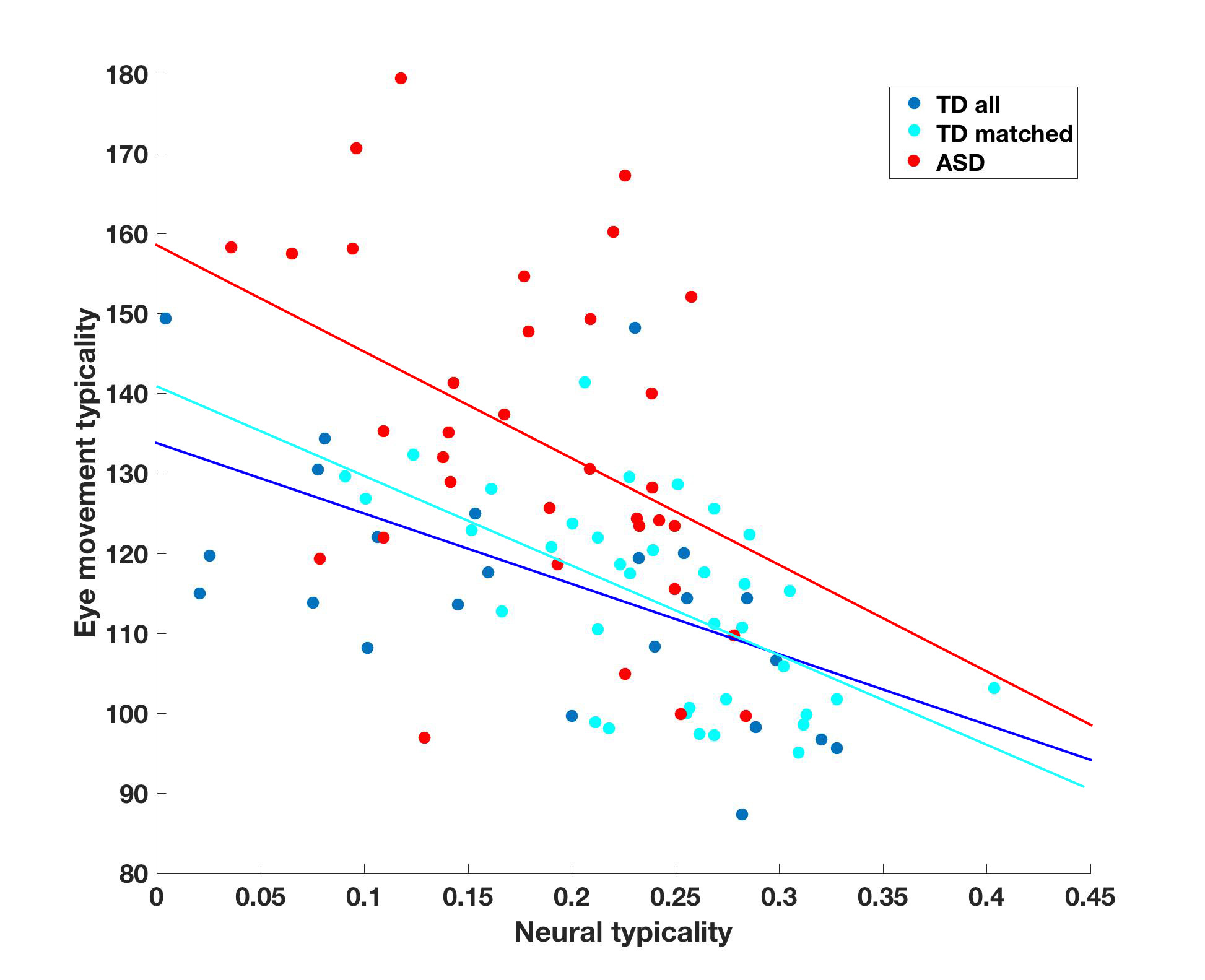
**

**Supplementary Figure 3: Correlations between neural typicality and eye movement typicality**. Correlation between eye movement typicality and neural typicality for the ASD group (red), averaged across the social orienting network as defined by the correlations of eye movement and neural typicality for the TD group. R = -0.42, p = 0.01. Correlations between the eye movement and neural typicality for the matched TD subset (cyan, r = -0.59, p = 1.6*10^-4^), and the entire TD group (remaining participants shown in blue, r = -0.56, p =4.2*10^-6),^ shown for context.

**
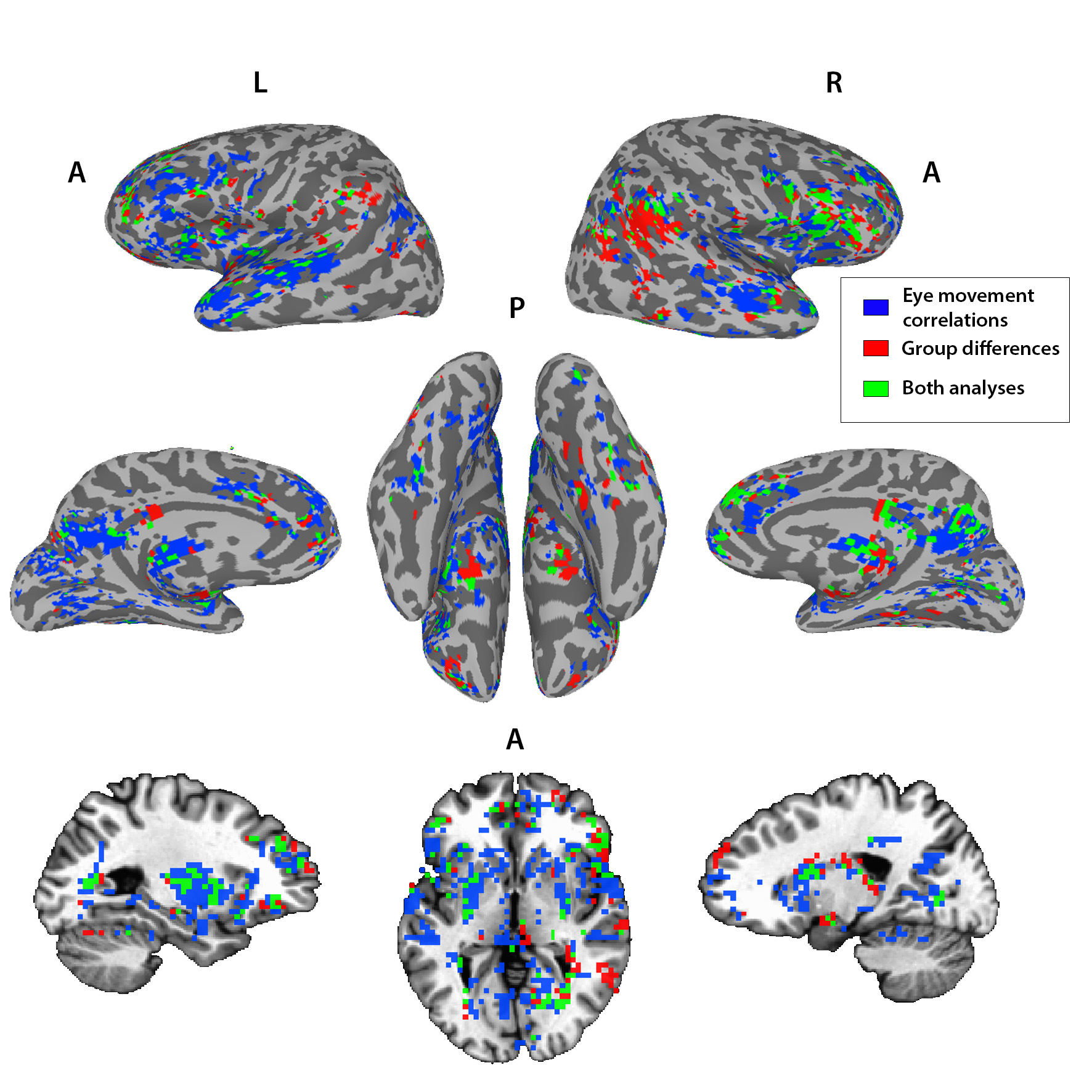
**

**Supplementary Figure 4: conjunction map of neural typicality group differences and eye movement correlations**. Overlay of the voxels with significant correlations between neural typicality and eye movement typicality of the combined matched TD and ASD groups (N = 36 TD + 33 ASD, blue), voxels showing significant group differences in neural typicality between the matched TD and ASD groups (N1 = 36 TD, N2 = 36 ASD, red), and voxels significant in both analyses (green). Threshold set at p<0.01, corrected for multiple comparisons through cluster size permutation tests.
