# Supplementary material for: Distinct neural mechanisms of social orienting and mentalizing revealed by independent measures of neural and eye movement typicality
Source: Commun Biol. 2020 Jan 29;3:48. doi: 10.1038/s42003-020-0771-1 (PMC6989525; doi:10.1038/s42003-020-0771-1)
Supplement: Supplementary file 2 — Reporting Summary [file 42003_2020_771_MOESM2_ESM.pdf]

natureresearch

Corresponding author(s): Michal Ramot

Last updated by author(s): Jan 3, 2020

Reporting Summary

Nature Research wishes to improve the reproducibility of the work that we publish. This form provides structure for consistency and transparency in reporting. For further information on Nature Research policies, see Authors & Referees and the Editorial Policy Checklist.

Statistics

For all statistical analyses, confirm that the following items are present in the figure legend, table legend, main text, or Methods section.

n/a	Confirmed	
☐	☒	The exact sample size (*n*) for each experimental group/condition, given as a discrete number and unit of measurement
☐	☒	A statement on whether measurements were taken from distinct samples or whether the same sample was measured repeatedly
☐	☒	The statistical test(s) used AND whether they are one- or two-sided<br>*Only common tests should be described solely by name; describe more complex techniques in the Methods section.*
☒	☐	A description of all covariates tested
☐	☒	A description of any assumptions or corrections, such as tests of normality and adjustment for multiple comparisons
☐	☒	A full description of the statistical parameters including central tendency (e.g. means) or other basic estimates (e.g. regression coefficient) AND variation (e.g. standard deviation) or associated estimates of uncertainty (e.g. confidence intervals)
☐	☒	For null hypothesis testing, the test statistic (e.g. *F*, *t*, *r*) with confidence intervals, effect sizes, degrees of freedom and *P* value noted<br>*Give P values as exact values whenever suitable.*
☒	☐	For Bayesian analysis, information on the choice of priors and Markov chain Monte Carlo settings
☒	☐	For hierarchical and complex designs, identification of the appropriate level for tests and full reporting of outcomes
☐	☒	Estimates of effect sizes (e.g. Cohen's *d*, Pearson's *r*), indicating how they were calculated

Our web collection on statistics for biologists contains articles on many of the points above.

Software and code

Policy information about availability of computer code

Data collection	MRI data was collected at the Functional Magnetic Resonance Imaging Core Facility on a 32 channel coil GE 3T (GE MR-750 3.0T) magnet and receive-only head coil. Eye movement data was recorded with the Eyelink 1000 Plus.
Data analysis	Post-hoc signal preprocessing was conducted in AFNI (Analysis of Functional Neuro-Images), Version AFNI_19.2.08 'Claudius', and data on the cortical surface were visualized with SUMA (Surface Mapping). Data were also analyzed with in-house software written in MATLAB (version R2016b).

For manuscripts utilizing custom algorithms or software that are central to the research but not yet described in published literature, software must be made available to editors/reviewers. We strongly encourage code deposition in a community repository (e.g. GitHub). See the Nature Research guidelines for submitting code & software for further information.

Data

Policy information about availability of data

All manuscripts must include a data availability statement. This statement should provide the following information, where applicable:

- Accession codes, unique identifiers, or web links for publicly available datasets
- A list of figures that have associated raw data
- A description of any restrictions on data availability

Data are available through NIH Figshare (10.35092/yhjc.c.4741556). All code will be available upon request. Unthresholded maps for the images depicted in Figures 3, 4, 5 and 7, as well as Suplemntary Figures 1,2 and 4are available through Neurovault: https://identifiers.org/neurovault.collection:6090

Field-specific reporting

Please select the one below that is the best fit for your research. If you are not sure, read the appropriate sections before making your selection.

☒ Life sciences ☐ Behavioural & social sciences ☐ Ecological, evolutionary & environmental sciences

For a reference copy of the document with all sections, see nature.com/documents/nr-reporting-summary-flat.pdf

Life sciences study design

All studies must disclose on these points even when the disclosure is negative.

Sample size	Optimal sample size is difficult to estimate in neuroimaging studies. However, the sample size here is comparable to or greater than what is usually reported in fMRI studies.
Data exclusions	One TD participant was excluded from the analysis because of an abnormal brain structure scan. 3 ASD participants and 2 TD participants failed to achieve adequate calibration with the eye tracker and were removed from the eye-tracking portion of the analysis
Replication	The reproducibility of the eye movement typicality rating was established through a split halves permutation test. The correlation between eye movement typicality and neural typicality was replicated across the TD and ASD groups
Randomization	No randomization was used, subjects were assigned to the ASD group based on clinical diagnosis
Blinding	Investigators were not blinded to group allocations, as these were determined by clinical diagnosis

Reporting for specific materials, systems and methods

We require information from authors about some types of materials, experimental systems and methods used in many studies. Here, indicate whether each material, system or method listed is relevant to your study. If you are not sure if a list item applies to your research, read the appropriate section before selecting a response.

Materials & experimental systems

n/a	Involved in the study	
☒	☐	Antibodies
☒	☐	Eukaryotic cell lines
☒	☐	Palaeontology
☒	☐	Animals and other organisms
☐	☒	Human research participants
☐	☒	Clinical data

Methods

n/a	Involved in the study	
☒	☐	ChIP-seq
☒	☐	Flow cytometry
☐	☒	MRI-based neuroimaging

Human research participants

Policy information about studies involving human research participants

Population characteristics	36 males aged 15-30 (mean age = 20.7) who met the DSM-IV criteria for autistic disorder, an autism cut-off score for social symptoms on the Autism Diagnostic Interview - Revised (ADI-R) and/or and ASD cut-off score from social and communication symptoms on the Autism Diagnostic Observation Schedule (ADOS), all administered by a trained, research reliable clinician, were recruited for the experiment. In addition, 63 typically developing participants (24 female) aged 15-30 (mean age = 22.05)
Recruitment	TD Participants were recruited through listserv announcements. ASD participants were recruited through referrals from Children's National Health Systems.
Ethics oversight	The experiment was approved by the NIMH Institutional Review Board (protocol 10-M-0027). Written informed consent was obtained from all participants or their guardians in the case of minors, in which case written assent was also obtained from the participants themselves

Note that full information on the approval of the study protocol must also be provided in the manuscript.

Clinical data

Policy information about clinical studies

All manuscripts should comply with the ICMJE guidelines for publication of clinical research and a completed CONSORT checklist must be included with all submissions.

Clinical trial registration	Clinical trials number NCT01031407

Study protocol	Full protocol can be accessed through https://clinicaltrials.gov
Data collection	Data were collected at the clinical center at the National Institutes of Health in Bethesda, Maryland. They were collected over an twenty month period, from May 2017 to January 2019.
Outcomes	N/A

Magnetic resonance imaging

Experimental design

Design type	Task
Design specifications	Free viewing of a 9.5 minute movie, no blocking
Behavioral performance measures	Participants were instructed to simply view the movie, no behavioral measures were recorded

Acquisition

Imaging type(s)	Functional, structural
Field strength	3 Tesla
Sequence & imaging parameters	The scans included a 5 minute structural scan (MPRAGE) for anatomical co-registration, with the following parameters: TE = 2.7, Flip Angle = 12, Bandwidth = 244.141, FOV = 30 (256 x 256), Slice Thickness = 1.2, axial slices. EPI scans were collected with the following parameters: TR = 2s, Voxel size 3*3*3, Flip Angle: 60, multi-echo slice acquisition with three echoes, TE1 = 17.5ms, TE2 = 35.3ms, TE3=53.1ms, Matrix = 72x72, slices: 28. 285 TRs were collected for the movie (9 minutes and 30 seconds). All scans used an accelerated acquisition (GE's ASSET) with a factor of 2 in order to prevent gradient overheating
Area of acquisition	28 slices did not quite cover the entire brain. The region was determined so that it captured as much as possible from the temporal pole upward

Diffusion MRI ☐ Used ☒ Not used

Preprocessing

Preprocessing software	AFNI (Analysis of Functional Neuro-Images), Version AFNI_19.2.08 'Claudius', and data on the cortical surface were visualized with SUMA (Surface Mapping)
Normalization	Data were transformed to Talairach space using a linear transformation, using AFNI's adwarp function
Normalization template	Data were normalized to original Talairach space
Noise and artifact removal	The first four EPI volumes from each run were removed to ensure remaining volumes were at magnetization steady state, and remaining large transients were removed through a squashing function (AFNI's 3dDespike). Volumes were slice-time corrected and motion parameters were estimated with rigid body transformations (through AFNI's 3dVolreg function). Volumes were co-registered to the anatomical scan. The data were then entered to a Multi-Echo ICA analysis (ME-ICA), as described in Kundu et al. 2013, to further remove nuisance signals (e.g., hardware-induced artifacts, residual head motion). Briefly, this procedure utilizes the physical properties of BOLD and non-BOLD fluctuations, namely the fact that whole signal from BOLD sources increases linearly over echo times, signals from non-BOLD sources remain constant across echoes. This allows the removal of non-BOLD fluctuations (noise).
Volume censoring	Data were not censored

Statistical modeling & inference

Model type and settings	N/A, data was treated like resting state data
Effect(s) tested	Pearson correlations between neural typicality (the correlation of the time course in each voxel for each participant with the average time course in that voxel across all other participants) was correlated with an independent measure of eye movement typicality (the correlation of the eye movement scan path for that participant with the average scan path across all other participants). Also, group differences in neural typicality between the TD and ASD groups were assessed using a two-tailed t-test.

Specify type of analysis: ☒ Whole brain ☐ ROI-based ☐ Both

Statistic type for inference (See Eklund et al. 2016)	Cluster-wise thresholds were determined through permutation based testing
Correction	The group analysis based maps in Figures 3 and 5 were corrected through FDR. The connectivity based maps in Figure 4,

Correction | were corrected through permutation based cluster size correction.

Models & analysis

n/a	Involved in the study
☐	☒ Functional and/or effective connectivity
☒	☐ Graph analysis
☒	☐ Multivariate modeling or predictive analysis

Functional and/or effective connectivity | Pearson correlation was used to determine functional connectivity
